# Cellists’ sound quality is shaped by their primary postural behavior

**DOI:** 10.1038/s41598-020-70705-8

**Published:** 2020-08-17

**Authors:** Jocelyn Rozé, Mitsuko Aramaki, Richard Kronland-Martinet, Sølvi Ystad

**Affiliations:** Aix Marseille Univ., CNRS, PRISM (Perception, Representations, Image, Sound, Music), 31 Chemin J. Aiguier, CS 70071, 13402 Marseille Cedex 09, France

**Keywords:** Skeleton, Acoustics

## Abstract

During the last 20 years, the role of musicians’ body movements has emerged as a central question in instrument practice: Why do musicians make so many postural movements, for instance, with their torsos and heads, while playing musical instruments? The musical significance of such ancillary gestures is still an enigma and therefore remains a major pedagogical challenge, since one does not know if these movements should be considered essential embodied skills that improve musical expressivity. Although previous studies established clear connections between musicians’ body movements and musical structures (particularly for clarinet, piano or violin performances), no evidence of direct relationships between body movements and the quality of the produced timbre has ever been found. In this study, focusing on the area of bowed-string instruments, we address the problem by showing that cellists use a set of primary postural directions to develop fluid kinematic bow features (velocity, acceleration) that prevent the production of poor quality (i.e., harsh, shrill, whistling) sounds. By comparing the body-related angles between normal and posturally constrained playing situations, our results reveal that the chest rotation and vertical inclination made by cellists act as coordinative support for the kinematics of the bowing gesture. These findings support the experimental works of Alexander, especially those that showed the role of head movements with respect to the upper torso (the so-called primary control) in ensuring the smooth transmission of fine motor control in musicians all the way to the produced sound. More generally, our research highlights the importance of focusing on this fundamental postural sense to improve the quality of human activities across different domains (music, dance, sports, rehabilitation, working positions, etc.).

## Introduction

Playing a musical instrument is an activity that involves complex auditory-motor interactions. Whether creating a short sound or developing a whole phrase, musicians must continuously establish a clear relationship between the actions *afforded* by their instrument and the auditory feedback resulting from their actions^1–3^. Research in neuroscience has demonstrated that such an active process intricately interweaves the auditory and motor regions of the brain as a neural substrate of cognitive representation^4,5^. In the case of the cello, for example, longitudinal studies conducted with non-musician participants and an MRI-compatible (Magnetic Resonance Imaging) instrument revealed that “brain plasticity” emerged as an integrative function of the neural network in auditory-motor information processing^6^. Both musical actions and percepts would thus depend on a single underlying mental representation governing both auditory encoding and motor control along the same goal-directed action. From these perspectives of embodied music cognition, we should consider the musical expressivity produced by instrumentalists as a link between sonic and corporeal movements and analyze their musical intentionality through the prism of a repertoire of learned gestural primitives^7,8^. Research in human biomechanics has highlighted that such a repertoire is composed of synergies, i.e., muscular cooperation patterns aiming to attain a given action^9^. A strong consequence of the synergetic mechanisms is that each voluntary action, such as moving a bow on a string, should be accompanied by anticipatory postural adjustments called APAs^10–12^. Anticipation is crucial in musical practice because of the coupling between coordination and postural balance, which implies that the fulfillment of a single goal-directed action may be encoded beforehand as a selective activation of the musicians’ joint degrees of freedom (DOFs)^13–15^. In dance practice, conversely, the mirror neuron system may decode the perceived expressiveness into fine movement structures through the same kind of grounded synergetic processes^16–18^. In the domain of rehabilitation, rhythmic auditory stimuli were efficient in reducing movement disorders and improving walking abilities in Parkinson’s disease and stroke patients^19–23^.

Due to the weight of teaching habits, ignorance or misunderstandings, the role of embodiment among musicians has been largely underestimated, despite evidence of its importance for the development of proficiency in many domains^24^. This underestimation is a recurrent problem in higher music education institutions that traditionally encourage the rapid acquisition of technical skills without sufficiently considering the development of musicians’ postural relations with their instruments^25^. Such pedagogical methods have always been the subject of heated debates and remain controversial today because of the high rates of dropouts due to psychological frustrations and musculoskeletal disorders among musicians^26^. Many students actually need to stop this *end-gaining* process and adopt alternative methods drawn from experimental psychology^27,28^, particularly the Alexander technique, which states that a well-directed *primary postural control*, i.e., a dynamic orientation of the head, neck, and upper back, has many benefits for coordination and musical expressivity^29^. Although very efficient in practice, this assumption has never been scientifically examined due to the difficulty of making accurate measurements of a musician’s primary postural control and of assessing its acoustical influence in an undisturbed way. In the area of bowed-string instruments, pioneer musical research analyzed sound features with bowing machines by focusing on physical control variables such as bow force and bow velocity but without considering the musician’s body^30–33^. Other studies assessed the instrumentalists’ auditory-motor mappings by means of motion and sound synthesis techniques with an electric violin^34–36^. More recently, psycholinguistic studies explored violinists’ cognitive processes by correlating perceptual adjectives of violin sounds (*round, harsh, light, mellow, dark, etc.*) to physical features of the acoustic signal and haptic feedback of the instrument^37–39^. Over the past two decades, we thus observed an increasing interest among the scientific community in better understanding the significance of musicians’ corporeal movements related to their expressive sound features. The results revealed the importance of such “ancillary” gestures in supporting or accompanying the instrumentalists’ “effective gestures” that are directly responsible for sound production^40–46^. In particular, investigations of clarinetists’ movements have shown that their sense of musical phrasing may be affected during ancillary impairment, i.e., when asked to move as little as possible while keeping their natural expressive intention or when the bell of their instrument was immobilized^47^. Such *disembodied* experimental conditions enable us to infer stable and reproducible patterns between musicians’ nonobvious movements and their audible components.

**Figure 1:**
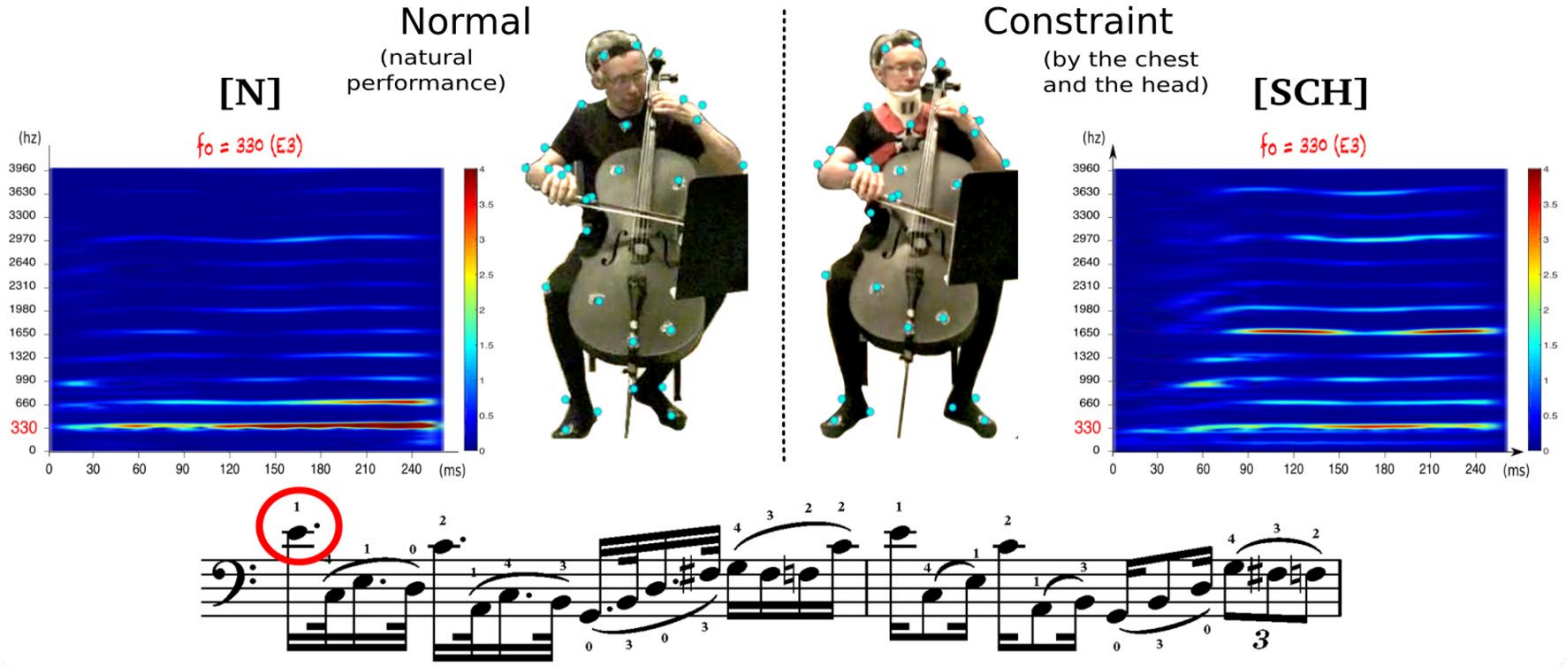
The musical passage and note investigated for this study. Spectrograms correspond to examples of the acoustic signal of an E4 note (the first one of this score sequence) played by the same cellist with good timbre quality (round) in the normal situation [N] and poor timbre quality (harsh) in the posturally-constrained situation [SCH] (Static Chest and Head).

In this study, we examined the key influence of musicians’ primary postural directions on their sound quality. This study is based on an experimental protocol^48^ that enabled us to compare the auditory-motor interactions of highly skilled cellists between two postural conditions: a natural condition and a posturally constrained condition in which the chest and the head were blocked by a safety race harness and a neck collar, respectively (cf “Methods” section). In the context of postural immobilization, the cellists’ timbre quality was consistently degraded on some key notes of the more demanding passages (cf Fig. 1). We supposed that this loss of expressiveness may correspond to specific deficiencies in the motor coordination of the right arm and impact the fluency, i.e., the level of precision, of the kinematic variations of the bow velocity. This assumption was inferred from the specialized literature on cellists’ physiology: the term bow “speed” can be used to describe the *degree of motor coordination* between the cellist’s body segments^49^; bow/string adherence, which shapes the timbre of the sound, would be more related to bow displacement than to bow pressure because no sound can be produced by only pressing the bow on a string without any movement^50^. We also built our experimental design on the assumptions provided by motor theories of perception^51–53^, that predict complementary relationships between nonverbal “gesticulations” in the case of speech and ancillary gestures in the case of music^54,55^. A psycholinguistic protocol actually revealed that inhibitions of nonverbal gestures caused speech to become much more laborious and tense, altering both intonation and expressiveness of the message^56^. This kind of connection was hypothesized in the music area through the existence of sonic-gestural objects, i.e., mental constructs in which auditory and motion elements co-occur both in the minds of the performer and the listener^57^. Such motor imagery of the musical experience would contain dyadic properties likely to activate linkages between the structure of the written score and esthetic concepts of the perceived sound^58,59^. According to this model, features that characterize the produced sounds may reveal the morphology of moving sonic shapes related to the kinematic displacements of the cellists.

**Figure 2:**
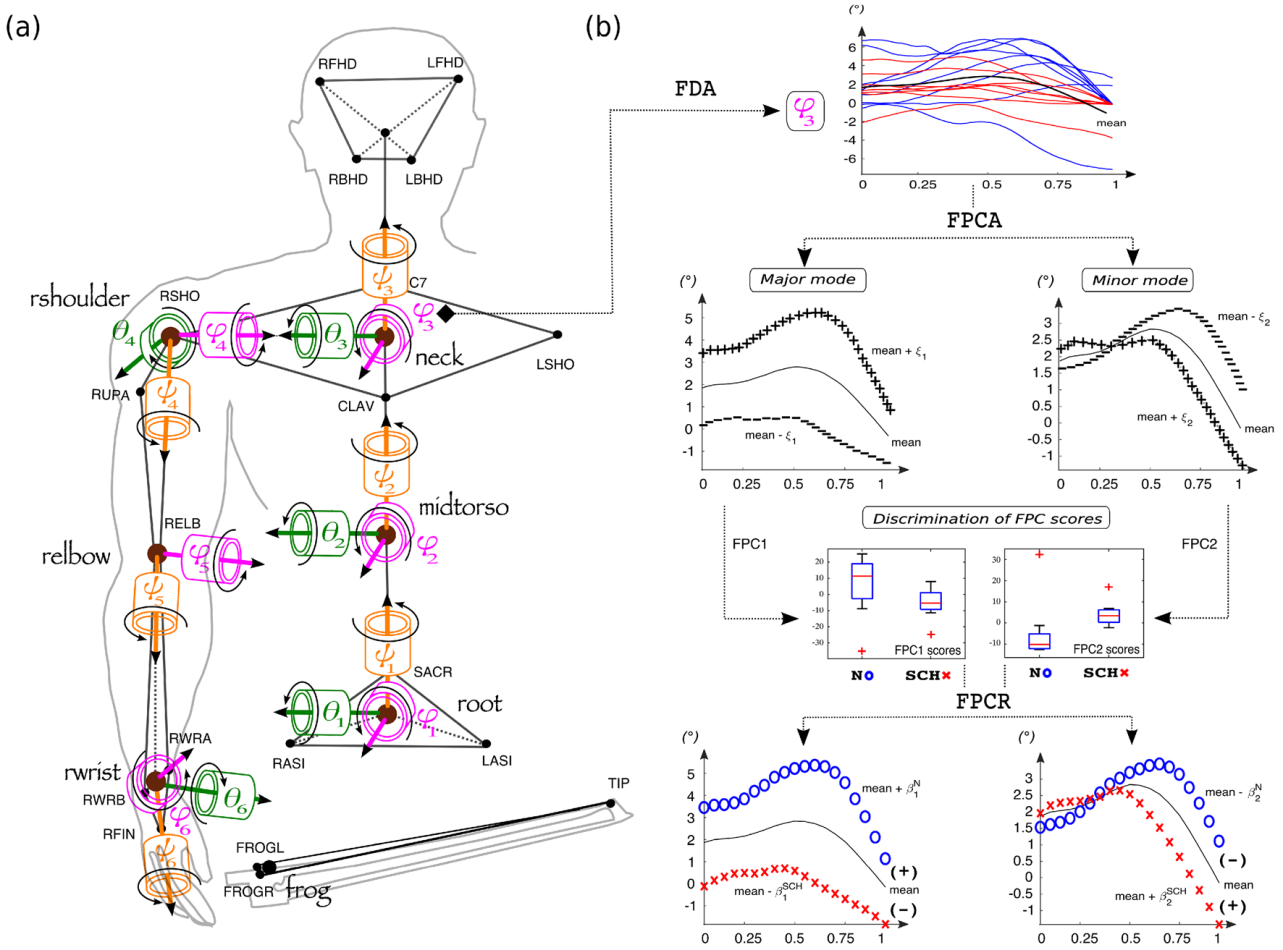
(**a**) Kinematic model of the cellists’ trunk and right arm bowing presented at rest (frontal view). This inertial system is composed of six key joints modeled as three single axes rotational joints in the Cardan/Euler angle representation {roll ($\psi _{\text {n}}$), pitch ($\theta _{\text {n}}$), yaw ($\phi _{\text {n}}$)} where $n\in [1\ldots 6]$ is the key joint number. (**b**) Statistical framework illustrated for a given anatomic variable of the kinematic model. This framework is based on functional principal component analyses (cf “Methods” section) and extracts two principal modes of variation of the cellists’ behavior, which are referred to as major mode and minor mode in the text. The effects of each mode are highlighted as functional deviations of the average time series between the normal (curves of blue circles) and the constrained situation (curves of red crosses).

Here, we chose to analyze cello sounds, commonly judged as poor or “harsh” in classical music, in terms of incorrect *moving sonic forms*^60^. In practice, this means that the acoustic signal variations analyzed within a harsh cello note may be expected to correlate with unsuitable bow velocity patterns, potentially induced from erroneous chest or head directions. Such sonic movements can be highlighted by crossing advanced methodological aspects of functional anatomy and acoustic processing (cf “Methods” section). Actually, movement scientists consider movement coordination the result of an organized motor activity, which can be divided into several elementary actions, also called *functional units*^61^. Similarly, psychoacousticians represent instrumental timbre within a perceptual space of several dimensions that are often related to temporal and spectral sound facets^62,63^. As cellists continuously modulate their gestures while playing, we may thus suppose that they use specific functional motion units to shape particular features of their sound production. This assumption guided us to design a statistical framework and to perform functional comparisons of the cellists’ kinematic and acoustic features between the normal and constrained conditions (cf Fig. 2b). The conception of this approach was inspired by research in the medical and biological engineering fields that provides efficient methods for comparing human motion patterns over time and for quantitatively emphasizing pathological deviations from a reference control group^64–67^. The results of those studies demonstrate that functional data analysis (FDA)^68^ and especially functional principal component analysis (FPCA)^69,70^ have better discriminatory power than the classical PCA multivariate approach^71^. FPCA is an emerging modern technique that extracts the principal modes (PCs) of a set of continuous waveforms and quantifies their differences across subjects as temporal deviations from the mean curve^72^. The technique has proven valuable for modeling simple motor behaviors^73–75^ or biomechanics of complex sport movements^76,77^, and in analyzing coarticulation patterns of musicians^78–81^ or spontaneous movement responses to music^54,82^.

In this study, we carried out functional PCA to determine the dominant components of the cellists’ audiomotor functional units and to assess their degradation on both the motion and the acoustic sides. The cellists’ bow velocity variations were defined as the main goal-directed actions, and the functional units set up to reach this goal were defined as the linear combinations of joint-related angular time series (cf Table 1). The acoustic variations were modeled by means of the descriptors highlighted in our previous work^48^ for characterizing the perceived harsh phenomenon (cf Table 2).

**Table 1:** Anatomic variables described as joint-related Euler angles {$\psi ,\theta ,\phi$} of the segmental kinematics.

Euler angle	Relation to segmental kinematics	
**Postural angles**		
root (1)		
$\psi _1$	Abdomen torsion	*To the left* [$0^{\circ }$...$+90^{\circ }$]
		*To the right* [$0^{\circ }$...$-90^{\circ }$]
$\theta _1$	Abdomen vertical inclination	*Forward* [$0^{\circ }$...$-90^{\circ }$]
		*Backward* [$0^{\circ }$...$+90^{\circ }$]
$\phi _1$	Abdomen lateral swing	*To the left* [$0^{\circ }$...$-90^{\circ }$]
		*To the right* [$0^{\circ }$...$+90^{\circ }$]
midtorso (2)		
$\psi _2$	Chest torsion	*To the left* [$0^{\circ }$...$+90^{\circ }$]
		*To the right* [$0^{\circ }$...$-90^{\circ }$]
$\theta _2$	Chest vertical inclination	*Forward* [$0^{\circ }$...$-90^{\circ }$]
		*Backward* [$0^{\circ }$...$+90^{\circ }$]
$\phi _2$	Chest lateral swing	*To the left* [$0^{\circ }$...$-90^{\circ }$]
		*To the right* [$0^{\circ }$...$+90^{\circ }$]
neck (3)		
$\psi _3$	Head torsion	*To the left* [$0^{\circ }$...$+90^{\circ }$]
		*To the right* [$0^{\circ }$...$-90^{\circ }$]
$\theta _3$	Head vertical inclination	*Forward* [$0^{\circ }$...$-90^{\circ }$]
		*Backward* [$0^{\circ }$...$+90^{\circ }$]
$\phi _3$	Head lateral swing	*To the left* [$0^{\circ }$...$-90^{\circ }$]
		*To the right* [$0^{\circ }$...$+90^{\circ }$]
$\psi _{12} = \psi _1+\psi _2$	Torso rotation	*To the left* [$0^{\circ }$...$+90^{\circ }$]
		*To the right* [$0^{\circ }$...$-90^{\circ }$]
**Instrumental angles**		
rshoulder (4)		
$\psi _4$	Upper arm rotation	*External* [$0^{\circ }$...$+90^{\circ }$]
		*Internal* [$0^{\circ }$...$-90^{\circ }$]
$\theta _4$	Upper arm abduction	*Abduction* [$0^{\circ }$...$+90^{\circ }$]
		*Adduction* [$0^{\circ }$...$-90^{\circ }$]
$\phi _4$	Upper arm anteversion	*Antepulsion* [$0^{\circ }$...$+90^{\circ }$]
		*Retropulsion* [$0^{\circ }$...$-90^{\circ }$]
relbow (5)		
$\psi _5$	Forearm rotation	*Supination* [$0^{\circ }$...$+90^{\circ }$]
		*Pronation* [$0^{\circ }$...$-90^{\circ }$]
$\phi _5$	Forearm extension	*Full flexion* [$0^{\circ }$]
		*Full extension* [$+180^{\circ }$]
rwrist (6)		
$\psi _6$	Hand rotation	*Supination* [+]
		*Pronation* [−]
$\theta _6$	Hand abduction	*Ulnar abduction* [$0^{\circ }$...$+90^{\circ }$]
		*Radial abduction* [$0^{\circ }$...$-90^{\circ }$]
$\phi _6$	Hand flexion	*Palmar flexion* [$0^{\circ }$...$+90^{\circ }$]
		*Dorsal extension* [$0^{\circ }$...$-90^{\circ }$]
$\psi _{56} = \psi _5+\psi _6$	Forearm rotation	*Supination* [$0^{\circ }$...$+90^{\circ }$]
		*Pronation* [$0^{\circ }$...$-90^{\circ }$]

The sign of each angle depends on its rotational direction that can be established from the resting kinematic model (cf Fig. 1a) by following the right-hand rule.

**Table 2:** Acoustic descriptors used in the study and their correlation to the perceived harshness phenomenon.

Name	Description	Correlation to harshness
HSV	Harmonic Spectral Variation^83^	Increase of harmonic asynchrony
ATS	Attack Time Slope^84^	Slower attack slope of the temporal envelope
MFCCratio	Ratio between MFCC coefficients c2 and c1^85^	Emergence of formantic area
SC	Harmonic Spectral Centroid^86^	Increase of spectral centroid
TRIratio	Ratio between tristimulus tr3 and tr1 + tr2^87^	Spectral energy transfer towards high-frequency components

## Results

**Figure 3:**
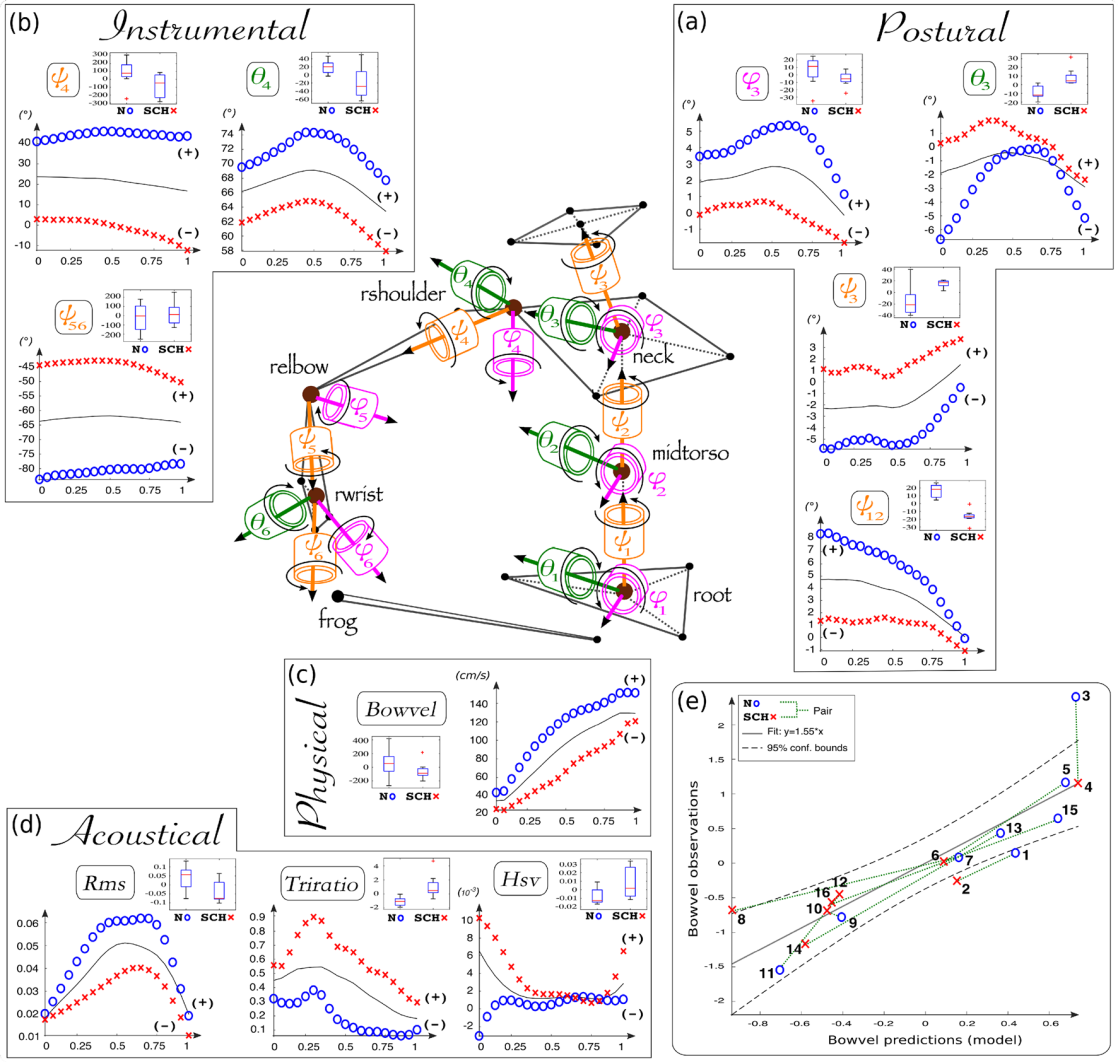
Major mode of the cellists’ functional variations. This mode explained 70% of the variance contained in the kinematic data—(**a**) postural, (**b**) instrumental, (**c**) physical—and 60% of the variance contained in the (**d**) acoustical data. At each stage of this functional unit, the effect of the major mode is visualized as functional deviations of the average time series between the normal situation (curves of blue circles) and the constrained situation (curves of red crosses). The attached boxplots present the distribution of FPC1 scores, i.e. the way each individual curve contributed to the major mode, for each variable that significantly discriminated the postural conditions. Normal and constrained functional components were added or subtracted to or from the mean curve, according to the mean sign of the FPC1 scores in each postural condition. The bottom right panel (**e**) shows the graph obtained by linear regression of the major scores (FPC1) of the bow velocity with respect to those of anatomic angles, which were significantly different between the postural conditions ($$R^{2}=0.90^{**}$$, $$R^{2}_{adjusted}=0.77^{**}$$).

**Figure 4:**
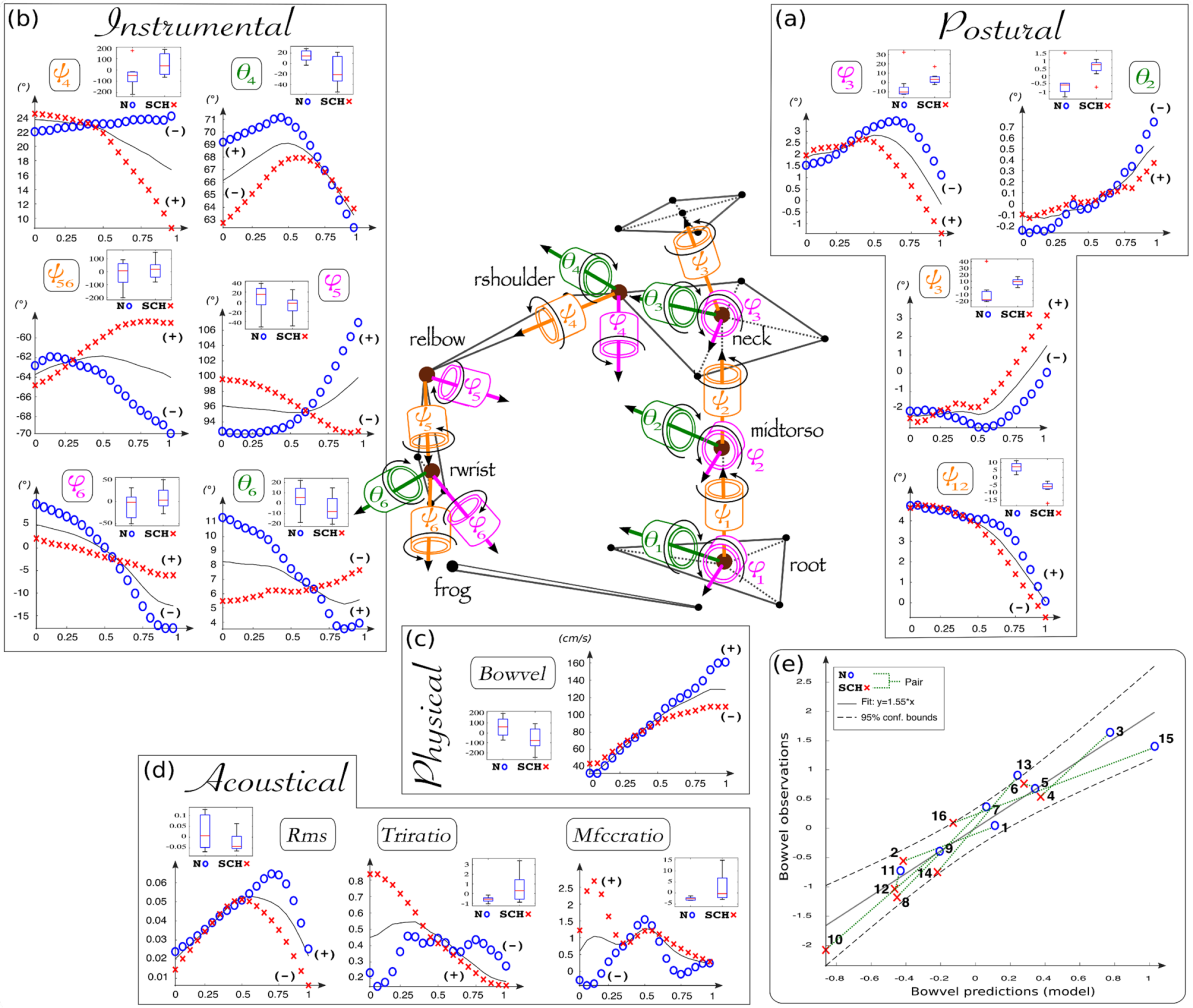
Minor mode of the cellists’ functional variations. This mode explained 25% of the variance contained in the kinematic data—(**a**) postural, (**b**) instrumental, (**c**) physical—and 15% of the variance contained in the (**d**) acoustic data. At each stage of this functional unit, the effect of the minor mode is visualized as functional deviations of the average time series between the normal situation (curves of blue circles) and the constrained situation (curves of red crosses). The attached boxplots present the distribution of FPC2 scores, i.e. the way each individual curve contributed to the minor mode, for each variable that significantly discriminated the postural conditions. Normal and constrained functional components were added or subtracted to or from the mean curve, according to the mean sign of the FPC2 scores in each postural condition. The bottom right panel (**e**) shows the graph obtained by linear regression of the minor scores (FPC2) of the bow velocity with respect to those of anatomic angles, which were significantly different between the postural conditions ($R^{2}=0.91^{*}$, $R^{2}_{adjusted}=0.73^{*}$).

By applying the steps of our analysis framework, which are thoroughly described in “Methods” section, we could infer two main functional auditory-motor linkages responsible for the perceived quality of cello sounds. In this paper, these two principal modes of variation are referred to as the major mode and minor mode. Each functional mode can be considered the coupling between an *eigenposture*^65^ and an *eigensonicform*^88^: an *eigenposture* describes a specific aggregate of postural and instrumental joint motions, and an *eigensonicform* describes a specific interaction of bow kinematics and acoustic features. FPCA analyses (cf Eq. 3) revealed that the major and minor eigenpostures captured approximately 95% of the total data variance, i.e., 70% for FPC1 and 25% for FPC2. Similarly, the major and minor eigensonicforms captured approximately 75% of the total data variance after smoothing, i.e., 60% for FPC1 and 15% for FPC2. Such percentages of the largest explained variance were sufficient to reveal the two most prominent timbre features and establish correlations with the kinematic behavior variations. Here, we present this *eigenfunction* structure with two figures describing the major mode (Fig. 3) and the minor mode (Fig. 4). For the sake of clarity, these figures only highlight the functional variables that presented significantly different behaviors between the normal and constrained situations.

### Major mode of variations

As observed in Fig. 3, the first (or major) functional unit corresponds to global amplitude variations at all the different stages of the sound-gesture chain. This was particularly salient at the physical stage, which reflects the “effective” sound-producing gesture (cf Fig. 3c), where the bow velocity globally decreased in the constrained condition (*Bowvel*: $t(7)=2.07$).

At the postural stage of the trunk motor chain (cf Fig. 3a), which reflects the ancillary gestures, this bowing alteration effect appeared to be associated with marked amplitude reductions of the natural chest torsion ($$\psi _{12}$$: $$t(7)=8.37^{***}$$) and head torsion ($$\psi _3$$: $$t(7)=-3.10^{*}$$). The analyses of the normal condition in the graph actually revealed surprising symmetrical evolutions towards zero for these two movements, with the chest torsion moving from the left and the head torsion from the right, while these tendencies were lost in the constrained condition. In accordance with Mantel^49^, we suggest that such a grounded tendency characterizes the need for a strong helicoidal energy transfer along the spine during the bow pulling movements to ensure optimal bow velocity amplitudes. The constrained condition also clearly affected the other degrees of freedom of the head, i.e., head elevation ($$\theta _3$$: $$t(7)=-3.42^{*}$$) and head lateral swing ($$\phi _3$$: $$t(7)=2.38^{*}$$), for which the amplitude variations were considerably smaller than their natural counterparts. Interestingly, these two analyses of the head under natural conditions in the graph revealed that the bouncing trend during the bow pulling movement, *up-and-down* and *right-and-left* was absorbed by the constraint.

At the instrumental stage (cf Fig. 3b), which reflects the interaction between effective and ancillary gestures, the major effect of postural impairments resulted in consistent amplitude alterations of the shoulder articulation, i.e., a loss of upper arm abduction ($$\theta _4$$: $$t(7)=4.40^{**}$$) and external rotation ($$\psi _4$$: $$t(7)=3.40^{*}$$). This insufficient upper arm external rotation also appeared to be symmetrically coupled to a loss of forearm pronation ($$\psi _{56}$$: $$t(7)=-2.32$$). Thus, in the constrained condition, the major mode reflects a systematic locking position of the whole right arm through unsuitably combined tendencies of upper arm internal rotations and forearm supinations that affected the bow velocity.

The results of multivariate regression on the major FPC scores of these anatomical angles was significant ($$R^{2}=0.90^{**}$$, $$R^{2}_{adjusted}=0.77^{**}$$, cf regression graph of Fig. 3e). It was therefore possible to infer a linear relationship predicting the global bow velocity amplitudes based on the set of anatomic angles selected by the first functional unit. More importantly, an additional stepwise regression extracted a combination of two angular degrees of freedom that explained the global variations of the bow velocity:

1 $$\begin{aligned} {\mathbf{Bowvel}} = 0.75 \times \psi _{2} - 0.52 \times \psi _{4} \end{aligned}$$

This simple predictive relation highlights a major mechanism of the cellist’s coordination, in which the coupling between the chest torsion (ancillary gesture) and the external rotation of the right arm (instrumental gesture) guaranteed suitable bow velocity amplitudes. More details on this major coordination mode (or *eigenposture*) could be obtained by computing correlations between the FPC scores. Interestingly, these results revealed that chest torsion was the coordinative support for bow velocity amplitudes ($$r_{\psi _{12}}^{Bowvel}=0.55^{*}$$). Further cross-correlations of the major angle scores revealed a chain of three coupling systems, which characterizes the coordination transfer within the major mode: (1) the system {$$\psi _{12}|\psi _{3}|\theta _{3}$$} showed the abovementioned symmetry of the chest/head torsions ($$r_{\psi _{12}}^{\psi _{3}}=-0.61^{*}$$); (2) the system {$$\psi _{3}|\theta _{3}|\phi _{3}|\psi _{4}$$} showed the importance of all the degrees of freedom of the head, especially of the head torsion, for activating the external rotation of the arm ($$r_{\psi _{3}}^{\psi _{4}}=-0.71^{**}$$); and (3) the system {$$\theta _{3}|\theta _{4}$$} showed that up-and-down head bouncing contributed to the amplitude of shoulder abduction ($$r_{\theta _{3}}^{\theta _{4}}=-0.53^{*}$$). No significant cross-correlations were obtained with the angle of prono-supination ($$\psi _{56}$$), which confirms that global bow velocity amplitudes were not controlled by the forearm but by the upper arm at the shoulder level through the helicoidal work of the trunk.

From the acoustical point of view (cf Fig. 3d), the major mode revealed that three of the five sound signal descriptors characterized the time-dependent perceptual differences between round and harsh cello sounds in terms of global amplitude variations. The graph analyses between normal and constrained situations revealed energy decreases within the temporal envelope (*Rms*: $$t(7)=3.04^{*}$$), energy increases on the upper partials of the spectral envelope (*Triratio*: $$t(7)=-3.53^{**}$$), and more harmonic asynchrony, especially during the birth phase of the sound (*Hsv*: $$t(7)=-2.08$$). More details about this major acoustic mode (or *eigensonicform*) could be obtained by computing correlations between the FPC scores. Surprisingly, these results revealed that the temporal energy level was the main descriptor impacted by global changes in bow velocity ($$r_{Rms}^{Bowvel}=0.52^{*}$$). More trivially, the cross-correlations of major acoustic scores revealed a strong collinearity between the amounts of harmonic asynchronicity and high-frequency spectral energy ($$r_{Hsv}^{Triratio}=0.75^{***}$$). No significant cross-correlations were obtained with the amount of temporal energy (Rms). These results suggest that the major functional coordination unit essentially captured the temporal variations of the sound shape responsible for harshness perception, independent of its purely spectral aspects.

### Minor mode of variations

As observed in Fig. 4, the second (or minor) functional unit corresponds to local variations of data amplitudes at the different stages of the sound-gesture chain. At the physical stage, which reflects the “effective” sound-producing gesture (cf Fig. 4c), the bow velocity decreased faster in the constrained condition than in the normal postural condition (*Bowvel*: $$t(7)=4.37^{**}$$).

At the postural stage (cf Fig. 4a), which reflects the ancillary gestures, this bowing deceleration appeared to be associated with a loss of natural bouncing between the chest torsion ($$\psi _{12}$$: $$t(7)=8.80^{***}$$) and the head torsion ($$\psi _3$$: $$t(7)=-2.35$$). The analyses of the normal condition in the graph actually revealed surprising symmetrical delays, chest torsion bouncing to the left and head torsion to the right, while these tendencies were lost in the constrained condition. In accordance with Hoppenot^25^, we suggest that such a grounded tendency characterized the need for a phase of active postural resistance to the bow pulling expansions to ensure optimal bow accelerations. This effect could also be observed in the lateral swings of the head ($$\phi _3$$: $$t(7)=-2.36$$) whose natural *right-and-left* bouncing disappeared in the constrained condition. Another interesting minor effect concerned the decrease in amplitude of the naturally vertical *down-to-up* inclinations of the chest ($$\theta _2$$: $$t(7)=-3.09^{*}$$) along the bow-pulling movements.

At the instrumental stage (cf Fig. 4b), which reflects the interaction between effective and ancillary gestures, the lack of an active resistance phase to the bow expansion was evidenced by the behavior of shoulder articulation through the loss of upper arm abduction ($$\theta _4$$: $$t(7)=3.39^{*}$$) during the beginning of the movement. The difference in external rotation was also very interesting ($$\psi _4$$: $$t(7)=-2.83^{*}$$) because it highlights the role of the shoulder in providing natural support that ensured the projection of the whole right arm. Actually, the amount of external rotation remained quite constant along a natural bow-pulling movement, whereas it drastically decreased in the constrained condition. It could also be observed that this naturally sustained external rotation guaranteed a reinforcement of the forearm pronation along the movement ($$\psi _{56}$$: $$t(7)=-2.05$$), whereas in the constrained condition, a forearm supination appeared as soon as the upper arm switched in internal rotation. Importantly, the minor mode of variations also revealed a strong difference in elbow flexion/extension between the two conditions ($$\phi _5$$: $$t(7)=3.54^{**}$$). In the normal condition, the elbow remained slightly bent during the phase of active resistance before it considerably stretched out during the phase of bow expansion. By contrast, in the constrained condition, the elbow increasingly flexed and locked the whole arm movement. This elbow-locking effect was also reflected by two losses of mobility at the wrist level: the *flexion-to-extension* progression ($$\phi _6$$: $$t(7)=3.48^{*}$$) and the *ulnar-to-radial* inclination ($$\theta _6$$: $$t(7)=2.38^{*}$$).

As for the major mode, the results from multivariate regression on the minor FPC scores of these anatomic angles were significant ($R^{2}=0.91^{*}$, $R^{2}_{adjusted}=0.73^{*}$, cf regression graph of Fig. 4e). It was therefore possible to infer a linear relationship predicting the local bow velocity amplitudes, or bow accelerations, based on the set of anatomic angles selected by the second functional unit. More importantly, an additional stepwise regression extracted a combination of two angular degrees of freedom that explained the local variations of bow velocity:

2 $$\begin{aligned} {\mathbf{Bowvel}} = 0.41 \times \theta _{2} + 0.64 \times \phi _{6} \end{aligned}$$

This simple predictive relation highlights a minor mechanism of the cellist’s coordination, in which the coupling between the vertical inclination of the chest (ancillary gesture) and the extension of the right wrist (instrumental gesture) ensured suitable bow accelerations. More details concerning this minor coordination mode (or *eigenposture*) could be obtained by computing correlations between the FPC scores. Interestingly, the results confirmed the importance of the vertical inclination of the chest ($$r_{\theta _{2}}^{Bowvel}=-0.58^{*}$$) and of the extension of the wrist ($$r_{\phi _{6}}^{Bowvel}=0.73^{**}$$) during bow accelerations. The scores of elbow extension were also marginally correlated to those of the bow accelerations ($$r_{\phi _{5}}^{Bowvel}=0.47$$,$$p=0.063$$). Further cross-correlations of minor angle scores revealed a chain of four coupling systems, which characterized the coordination transfer within the minor mode: (1) system {$$\theta _{2}|\psi _{12}|\psi _{3}|\phi _{3}$$} showed the postural coupling among the chest torsion and vertical inclination ($$r_{\theta _{2}}^{\psi _{12}}=-0.51^{*}$$), the bouncing symmetry of chest/head torsions ($$r_{\psi _{12}}^{\psi _{3}}=-0.56^{*}$$), and the strong dependence between head torsions and lateral swings ($$r_{\psi _{3}}^{\phi _{3}}=0.90^{***}$$); (2) system {$$\psi _{3}|\phi _{3}|\theta _{4}|\psi _{4}|\psi _{56}$$} showed the importance of the degrees of freedom of the head, especially of the head torsion, to activate the external rotation of the arm ($$r_{\psi _{3}}^{\psi _{4}}=-0.71^{**}$$) and that of the coupling between this external rotation and the forearm pronation ($$r_{\psi _{4}}^{\psi _{56}}=0.52^{*}$$); (3) system {$$\psi _{12}|\psi _{3}|\phi _{3}|\theta _{6}$$} showed the indirect influence of many postural angles, especially the angles linked to head torsion and lateral swing on the wrist inclination ($$r_{\psi _{3}}^{\theta _{6}}=-0.64^{**}$$ and $$r_{\phi _{3}}^{\theta _{6}}=-0.66^{**}$$ respectively); and (4) system {$$\phi _{5}|\phi _{6}$$} showed that the wrist extension was conditioned by the elbow extension ($$r_{\phi _{5}}^{\phi _{6}}=0.71^{**}$$). These results confirmed the importance of the double phase of postural resistance/expansion along the movement for ensuring optimal bow pulling accelerations.

From the acoustical point of view (cf Fig. 4d), the minor mode revealed that the same acoustic descriptors as in the major mode with an additional fourth descriptor, the Mfccratio, were significantly affected by the constrained condition. The analyses in the graph revealed an inability to maintain the acoustic signal energy during the entire movement in the constrained condition. This effect was noticeable both in the temporal domain and in spectral domains (*Rms*: $$t(7)=2.08$$, *Triratio*: $$t(7)=-2.45^{*}$$, respectively). In particular, the Mfccratio revealed an excessive amount of high-frequency spectral energy at the beginning of the sound that corresponded to the emergence of a formantic area (*Mfccratio*: $$t(7)=-2.04$$). More details concerning the minor acoustic mode (or *eigensonicform*) could be obtained by computing correlations between the PC scores. Surprisingly, the results revealed that the amount of high-frequency spectral energy was the main descriptor impacted by local changes in bow velocity ($$r_{Triratio}^{Bowvel}=-0.58^{*}$$). More trivially, the cross-correlations of minor acoustic scores revealed a strong collinearity between the amounts of spectral energy ($$r_{Triratio}^{Hsc}=0.88^{***}$$) and formantic energy ($$r_{Triratio}^{Mfccratio}=0.60^{*}$$). No significant cross-correlations were obtained with the amount of temporal energy (Rms). Complementary to the major mode, these results suggested that the minor functional coordination unit had essentially captured the variations in the spectral shape of the sound responsible for harshness perception (independent of its temporal aspects).

## Discussion

In summary, our functional analyses revealed that two primary postural directions are involved in the sound quality produced by highly skilled cellists: first, a major mechanism controlling bowing velocity (cf Eq. 1) linked to the evolution of the temporal shape of the sound and, second, a minor mechanism controlling bowing acceleration (cf Eq. 2) linked to the evolution of the spectral content of the sound. These results are consistent with the physics of the instrument and the pioneering acoustic studies based on bowing machines. First, the bow velocity should be correlated to the amount of transmitted vibrations to the surrounding air by the body of the cello and thus determine the energy level or intensity of the acoustic signal. Actually, harsh sounds correspond to global decreases in bow velocity and weaker temporal profiles of acoustic energy (cf major mode of Fig. 3c,d). Second, the bow acceleration should be correlated to the amount of high-frequency energy and thus determine the quenching rates of upper partials in the spectrum^89^. Harsh sounds actually correspond to global bow decelerations and higher quenching rates of spectral energy for upper partials (cf minor mode of Fig. 4c,d). Among the set of acoustic descriptors that characterize perceived harshness, harmonic asynchrony remains only poorly explained by kinematic bowing analyses. This indicator of spectral fluctuations might be influenced more strongly by the strict bow/string adherence finely tuned by the bow force parameter^90^. As a perspective, it may thus be interesting to reiterate the same kind of functional analyses with dynamic features, i.e., the prediction of variations in bow force from the muscular efforts estimated for each cellist’s body segment. Nevertheless, the bow/string adherence quality involved in the perceived sound *density* depends on the bow velocity^50^, which was insufficient in this study when the right arm remained locked in a position of excessive supination and internal rotation.

From our coordination study, such a tighter instrumental bowing gesture would be caused by inadequate combinations of postural variables, particularly the loss of a symmetric combination between chest and head torsion movements. The freedom of the head movement was particularly important to balance the chest torsion with the external rotation of the right arm involved in both kinetic functionalities of the bowing (velocity and acceleration). These results are consistent with previous studies on cellists’ right arm behaviors, especially the role of shoulder mobility during musical playing on the A string^91,92^. Furthermore, our findings emerged from large bow pulling gestures on one note, for which the impaired cellists could not compensate as simply as elsewhere in the score. As the chest and head constraints affected the cellists’ sound quality on other notes to a lesser extent, the execution of this particular note would stand for a limit in terms of postural adaptation, which clearly depends on the score structure and not only on the ergonomics of the instrument. By generalizing to the whole score, we suggest that this salient local effect of recurrent sound degradation highlights a more generic deficiency of cellists’ postural control, also called *posturo-kinetic capacity*^12^ in movement science. Even though its variations may remain subtle, such a capacity would guarantee body stability during any goal-directed action, such as bowing on one or several notes. Actually, the postural deviations of our highly skilled cellists were no more than 5 degrees from the mean value in the major mode (cf Fig. 3a), but this was enough to globally influence the quality of their auditory-motor interactions. This postural capacity is also highlighted through a double phase of postural resistance/mobility to bow expansion in the minor mode (cf Fig. 4a), which resulted in spectral alterations in the sound when the musicians were posturally impaired. The constraints thus revealed the cellists’ primary postural directions by *disembodiment*^8^, which supports the idea that the musicians’ structural and expressive concepts are grounded in their sensorimotor networks.

The correlations established between the cellists’ movements and their sound quality features also provide knowledge on their theoretical physiological principles^49,93^. Actually, our results suggest that the cellists’ bowing actions would be more effective if organized in terms of “distal events”^94,95^, i.e., when their attention is not centered on the movement itself but more on its potential influence on the sound quality. Here, we suppose that the postural impairment considerably disrupted the musicians’ natural sensations, i.e., the external focus of attention needed to correctly perform an expressive musical task (professional cellists often talk about “playing without thinking”). As such, the context of this experiment may be considered a relevant “constrained action hypothesis”^96,97^ for reinforcing the concept of *supra-postural activity*^98,99^: the quality and efficiency of a task would depend on this supra-postural control, i.e., the way individual body movements are subsumed into a unified Gestalt for achieving the given goal. Interestingly, two of the seven cellists in our experiment stated that they became more aware of their belly respiration in situations of postural impairment. In our opinion, these remarks indicate that before being impaired, both respiratory and postural control were naturally piloted by an external focus, i.e., by supra-postural commands of their attention. The constraint forced the cellists to adopt an internal focus and to compensate by more conscious control of their movements. We hereby consider that these scientific deductions give strong support to the concept of *primary postural control*, which was postulated as part of the Alexander technique^100^, not only in the context of instrumentalists but also for any goal-directed actions requiring a strong supra-postural activity. By encouraging performers to focus on the results of the actions rather than on the actions themselves, the motor system could be trained in a more embodied and self-organized way for natural and efficient performances.

These findings clearly suggest important applications for improving and optimizing practice habits among musicians. This subject is a hot topic in research areas that assess the risk of musculoskeletal disorders among musicians and search for strategies to promote health or reduce injury^26,101,102^. Feedback analyses of students in higher music education institutions especially revealed the upper limb, upper trunk, and neck as the main body parts affected by muscle pain syndromes^102–104^. The population of bowed-string players would also be more affected by these postural disorders because of the asymmetric arm positions related to the trunk^105,106^. Such results are clearly compliant with those of our study and reinforce the importance of integrating musicians’ primary postural control within individual rehabilitation programs. The magnitude of the cellists’ spinal curvatures that we highlighted in relation to their sound quality may particularly help in developing strengthening-flexibility exercises targeting the trunk muscles of bowed-string players. As a whole, we think that the constrained condition of our experiment altered the natural musicians’ action/perception cycle in a way that could be referred to as a “phenomenological experience on non-sense”^107^. If cognition is our way of dealing with non-sense experiences, then the tools established to reeducate the musicians’ proprioceptive feedback should authorize such an experience on nondoing or nonactivity consciousness, also known as *inhibition* in the Alexander technique^29^. We hereby support the idea that the quality disruptions occurring in a musical discourse find their origin in a *faulty postural awareness*^100^, and may be solved by refining the musicians’ global perception of somatosensory processing.

The findings presented in this paper may also have a strong impact in other areas related to expert performance, especially due to the statistical framework that we established. Sports biomechanics is one example of a domain where body posture, dynamic somatic practice, and motor control need to remain inherently and strongly connected to ensure the efficiency of a given action^66,76,77,108^. For example, research on human-material interfaces demonstrated that tennis players or runners need to finely tune the shock vibrations induced by the racket or the ground surface^109–111^. In that context, functional data analyses may provide an opportunity to infer continuous patterns of adaptation between the effector limb (hand or foot) and the entire body of these athletes. By extension, such analyses could also highlight a functional interdependence between the sound produced in reaction to the impact (with a racket/ground surface) and the biomechanical propagation of shock-induced vibrations. Such examples suggest that our statistical framework may be suitable for analyzing the sound-gesture relationships in a reverse way, i.e., assessing the role of auditory information on perceptual-motor processes. In recent years, many studies have highlighted the benefits of *gestural sonification*^5,112–115^, especially in the domains of sports performance and motor rehabilitation^116,117^. For example, sonification efficiently reduced the variability of golf swing gestures in novices^118,119^, or improved the pedal force effectiveness among cyclists^120^. The beneficial effects of sonification in reeducating patients with severe gait dysfunctions, such as Parkinson’s disease patients, by rhythmic auditory cueing^21–23,121^, or neuromotor deficits related to the fluency of handwriting, such as dysgraphia^122–126^, were also well recognized. In the same way, we suppose that such continuous auditory feedback may help musicians and dancers improve or recover their body awareness, for example, through experiments of *sound tracing* and motor mimicry, which are already known to stimulate covert mental images associated with musical experience^58,81,127,128^.

## Conclusion

In this paper, we assessed how postural impairments of highly skilled musicians affected their perceived sound quality. Through functional analyses of cellists’ kinematic and acoustic interactions, it could be demonstrated that feedforward deficiencies of the primary postural command locally altered the quality of their musical expression. Such findings suggest that musical teaching should, to a much greater extent, consider the student?s body as a global flexible and proactive structure rather than focusing on specialized cognitive patterns that break the sensorimotor processes into rigid units. This conclusion is consistent with embodied learning frameworks, especially the Alexander technique, that correlate optimal body usage to proper directions of the spinal structure and fine balance mechanisms between the head, neck, and trunk. It should therefore be possible to influence expressive perceptual processes and thus shape the musical mind by developing a kinesthetic awareness of the sensory-motor relationships, i.e., integrating the sensations of joint mobility, muscular stability, and posture as a whole. If such *indirect procedures* would contribute to reinforcing musculoskeletal health and the quality of the performance in the musical domain, they may also be applied in a reverse way for learning dance and sport skills or for patients in clinical rehabilitation by means of experimental manipulations of auditory feedback.

As a promising perspective of this study, we started to develop a complementary approach for assessing the effects of harsh timbre degradation on cellists’ motor behavior. By means of our statistical framework of functional analyses, we expect to close the perceptual loop that links cellists’ timbre quality to their postural control. The methodological aspects of such a work are based on the use of an electric silent cello and the setup of a multimodal platform combining a motion capture system and spatial rendering to study sound/gesture interactions. We think that augmenting the perceptual information, especially through fine sound synthesis techniques applied to gestural sonification, might provide a suitable means to strengthen the understanding of the *body schema* related to cognitive interpretation and physical expression of structures within music or dance performance. Such an approach has the potential to guide research on the design of skill training or rehabilitation scenarios in the context of real-world applications, and it is particularly well-suited for (but not limited to) musicians and dancers.

## Methods

### Participants

Seven highly skilled cellists (males = 4; females = 3; mean age = $40.5\pm 11.1$) were recruited on a voluntary basis from the Music Conservatory and the Opera of Marseille to participate in a 3-h experiment that, as they were told, consisted of‘ “exploring cellists’ sound/gesture relationships”. Before the experiment, each musician signed a consent form that advised them of the precise the nature of the postural conditions and in which they agreed to the publication of the information/image(s) collected during the experiment in an online open-access publication. The musicians were also given an honorarium for their participation. All the procedures of the protocol were approved by a local ethics board at the ISM-Aix-Marseille University and were carried out according to the relevant guidelines expressed in the 1964 Declaration of Helsinki.

### Design and apparatus

The design of our experiment was based on four postural conditions of gradual difficulty^129^. For each condition, the cellists were asked to play a score composed of different technical patterns as expressively as possible. The full score was executed three times by postural condition, according to two tempi [45/70 bpm] and bowing modes [*detached/legato*]. The postural conditions and repetitions of factor combinations were randomly presented to each participant. At the end of each postural session, we collected the participants’ impressions regarding their difficulties in terms of motion and sound production by means of a short questionnaire. In this paper, we focus on the two extreme experimental conditions (cf Fig. 1): the natural performance (entitled [N]: *Normal*) and the fully constrained condition (entitled [SCH]: *Static Chest and Head*). This fully constrained condition consisted of impairing the cellists by two immobilization devices that reduced their primary postural control in a noninvasive way: a six-point safety race harness that restrained the torso displacements and an adjusted neck collar that limited the freedom of head movements. We installed this equipment on the musicians so that their shoulder mobility was not affected.

The cellists’ movements were recorded by an infrared motion capture system (Vicon 8, fps=125 Hz) that tracked the three-dimensional positions of the reflective markers positioned on the performer’s body and the instrument. We followed the anatomical “Plug-in-Gait” (Vicon Motion Systems. Plug-in-Gait product guide. Oxford: Vicon Motion Systems, 2010, https://www.c-motion.com/download/IORGaitFiles/pigmanualver1.pdf) standard to distribute the marker locations on the instrumentalist’s body. For this study, we focused the kinematic analyses on a subset of seven key markers covering the cellists’ postural chain (torso/head) and the instrumental chain responsible for the bowing gestures produced by the right arm. Some of these markers were virtually computed from the Plug-in-Gait anatomical landmarks located on each segment, in accordance with the Dempster model convention^130^ (cf Supplementary Table 1). The acoustic signals produced by the instrument were recorded by a DPA 4096 microphone placed under the cello bridge and connected to a MOTU interface (Ultralite MIC3, fps = 44.1 kHz). Both recording systems were synchronized by a manual clap.

### Stimuli and procedure

The stimuli were extracted from the cellists’ post-experimental feedback, which identified a part of the performed score as frequently degraded in the constrained postural situation. Actually, several notes belonging to this passage sounded harsher and shriller in agreement with the cellists’ comments regarding their performance, in particular, their impression of producing “tighter and tenser sounds”, or “sounds lacking depth and natural resonance”. Such harshness phenomena (i.e., degraded, metallic sound color) occurred during the execution of quick syncopated patterns requiring excellent synchronization between the two arms and were quite consistent among cellists on the first note of the sequence (cf Fig. 1). This dotted sixteenth of pitch E4 is a key note that provides the motion impulse to the musical phrase through a large bow-pulling gesture on the first (A) cello string. Spectrogram analyses of this note between the normal and constrained postural situations revealed salient signal differences, which were thoroughly explored and connected to the musicians’ perception in a previous work^48^. We assessed the qualitative harshness phenomenon judgments according to esthetic criteria of classical music by means of perceptual tests administered to a population of 15 trained cellists, both teachers and advanced students. None of these cellists had participated in the experiment and had no knowledge of the constrained postural conditions.

For this paper, we used the same corpus as in our previous work, which was built from perceptual evaluations of harshness between the normal and constrained performances of the seven cellists. This corpus was composed of the eight most salient pairs of round/harsh (good/poor quality) sounds of the E4 note, extracted from the cellists’ performances in the normal and constrained contexts (mean note duration = $310\pm 60$ ms $\forall$ [N/SCH]). Each round[N]/harsh[SCH] note pair belonged to a given cellist performing in slow tempo (45 bpm) and *legato* bowing mode. The pairs of samples also belonged to different cellists and could thus be considered independent.

### Motion analyses

Motion analyses were based on the anatomic displacements of the cellists’ joints associated with each sound of the corpus and on their bow velocity over time. To assess fine coordination features, we designed a kinematic model describing the temporal evolution of these body joints (cf Fig. 2a). The model was composed of a linkage of six main rotary joints (cf Table 1) articulating seven segments related to the body trunk and the right arm (pelvis, abdomen, chest, head, upper arm, forearm, hand). Each corporeal segment was assumed to be a rigid link, and the six articulations were approximated from the skeleton geometry as spherical joints of three-dimensional degrees of freedom (DOFs)^131,132^. We computed 18 DOFs (6 joints $\times$ 3 angles) as joint-related triplets of anatomic angles {$\psi _n,\theta _n,\phi _n$}, $n\in [1\ldots 6]$ (cf Table 1) by performing Cardan/Euler conversions of their segment-related marker coordinates^132,133^. For each joint, the method consisted of computing the way the distal segment of the join was spatially rotated with respect to its proximal segment (cf Supplementary Figure 1). In geometric terms, this approach merely defined a rotation matrix between two bases {$\vec {i}^{p},\vec {j}^{p},\vec {k}^{p}$} and {$\vec {i}^{d},\vec {j}^{d},\vec {k}^{d}$} attached to the joint proximal and distal segments, respectively (cf Supplementary Table 2). Such a matrix represents a succession of three rotations needed to transform a joint proximal basis into its relative distal basis: first rotation around X by an angle $\psi$ (*roll*), second rotation around Y by an angle $\theta$ (*pitch*), and third rotation around Z by an angle $\phi$ (*yaw*). As six rotation matrices should be computed to model all the DOFs, we iterated the process along the six reference body hinges of the cellists’ motor chain. In addition to these joint single-axis rotations, we also defined two composite angles for characterizing the global torso rotation ($\psi _{12}$) and the global forearm pronation/supination ($\psi _{56}$). Note that angle $\theta _{5}$ was removed because of the redundancy with the external/internal rotation of the shoulder ($\psi _{4}$); most biomechanics literature actually expresses the elbow joint by only two DOFs: flexion/extension ($\phi _{5}$) and pronation/supination ($\psi _{5}$)^134^. At the end of this chain of anatomic angles, the bow velocity was computed as the velocity vector norms of the bow “frog” marker (cf Supplementary Table 1) along the duration of each note composing the corpus: $Bowvel = \sqrt{(v_x^2+v_y^2+v_z^2)}$, where the triplet ($v_x,v_y,v_z$) refers to the derivatives of the spatial coordinates of the bow frog at a given time.

### Acoustic analyses

Acoustic analyses were based on the computation of five acoustic descriptors over time (cf Table 2), which had been determined to be significant in our previous work^48^ for discriminating between round and harsh cello sounds. The extraction process for note E4 relied on a pitch-tracking algorithm adapted from the MIR toolbox (Music Information Retrieval)^135^ of MATLAB software. We developed a dedicated workflow in MATLAB to compute the five acoustic descriptors over time by following the MPEG-7 standards^136^: HSV (Harmonic Spectral Variations) relates to the sound spectral flux as a time-varying spectral content of its harmonic components^83^; it was obtained from the spectral variation of harmonic amplitudes between adjacent temporal frames. ATS (Attack Time Slope) corresponds to the attack time slope of the sound signal; it was determined from the logarithmic rise time of the signal energy during the attack phase. MFCCratio is a ratio between the two first MFCCs (Mel-Frequency Cepstral Coefficients)^85^, which we designed to highlight specific variations of the sound spectral envelope in a perceptual way; the coefficients were classically obtained through a DCT (Discrete Cosine Transform) applied to the logarithmic spectral envelope. SC (Spectral Centroid) corresponds to an amplitude-weighted mean of the harmonic spectral peaks; it was obtained through a decomposition in subbands centered on the signal harmonics^137^. TRIratio describes the spectral energy distribution in three frequency bands as an energy ratio between each band and the total number of harmonics. The first band contains the fundamental frequency, the second band contains the medium partials (two, three, four) and the last band contains the higher partials (five and more). The three tristimulus coordinates were obtained by spectral centroid computations for each band^87^.

### Statistical framework

Our statistical framework was designed with the aim of carrying out functional comparisons of the cellists’ sound-gesture interactions between the normal and constrained conditions. This process can be divided into five steps, which are described below by referring to the schema components of Fig. 2b. All the calculations were performed with the help of MATLAB software and the FDA toolbox^138^.

#### Functional data analyses (FDA)

In contrast with the classic PCA approach, functional data analysis considers the entire sequence of measurements a function or a single entity rather than a series of individual data points^76^. To represent our motion features (anatomic angles, bow velocity) and acoustic descriptors as time-varying functions, the FDA methodology consisted of decomposing each time-series of variables as a linear combination of B-spline basis functions. We chose an equally spaced 6-order B-spline basis because it was better suited for numerical calculations than polynomials that are less stable. Furthermore, B-spline functions were very useful for smoothing acoustic data of noisier natures than kinematic data while efficiently accommodating changes in local behavior. A semi-sampled spline basis was sufficient to keep a fine-grained definition of each curve. The B-spline mathematical decomposition is also required to align the $n=16$ time series (eight {normal/constraint} data pairs) of motion and acoustic descriptors to the duration of the longest series beforehand. This duration was normalized between 0 and 1 to be consistent with the FDA time-warping mechanism.

#### Functional principal component analyses (FPCA)

FPCA was carried out based on the spline-based representation of time-point data. This technique has the major advantage of producing functional principal components that can be interpreted in the same domain as the original observations (kinematic and acoustic). Actually, this technique models each descriptor time-series $f_i$ as a linear combination of weighted deviations from its mean dataset $\overline{f_i(t)}$:

3 $$\begin{aligned} f_i(t) = \overline{f_i(t)} + \sum _{k=1}^{K} c_{ik} \xi _k(t) + \epsilon _i, \; c_{ik} = \int \xi _k(t) f_i(t) dt \end{aligned}$$

where $\xi _k(t)$ are the functional principal components (FPCs), also called *eigenfunctions*, that captured the *K* first main hidden modes of variations. The coefficients $c_{ik}$ correspond to score projections as in classical PCA but assess the extent to which the shape of each individual behavior $f_i$ of the dataset matches with the global mean trend $\overline{f_i(t)}$. $\epsilon _i$ is the prediction error between the observations $f_i(t)$ and their model as a sum of projections on the *K* principal modes.

In this study, for each kinematic or acoustic descriptor, we performed an FPCA on the set of its spline-based time-series $f_i(t),i \in [1,16]$, without considering, for the moment, a separation between the normal and constrained conditions. The acoustic descriptors were processed by adding a small amount of smoothing to the B-spline model to more easily capture the main variation trends while avoiding distortion of the data. The deviation patterns obtained by FPCA, especially those related to the acoustic descriptors, took into account this compromise between data smoothness and the largest proportion of explained variance. According to the statistics literature^68,70^, FPCA should be interpreted through graphs that present the ensemble mean curve of the original observations ($\overline{f_i(t)}$) and the functions obtained by adding or subtracting a suitable multiple of each FPC ($\xi _k(t)$) to or from this mean. Generally, this multiple corresponds to the percentage *p* of explained variance, which can be written in this way: $\overline{f_i(t)} \pm p \times \xi _{k}(t)$. We followed this methodology in the paper to explain the $K=2$ main modes of variation resulting from our analyses through two figures describing their detailed effects on both motion and acoustic sides (cf Figs. 3 and 4). In these graphs, the decision to add or subtract a functional component to the mean curve was made according to the mean sign of the FPC scores of each postural condition.

#### Statistical comparisons of the functional principal components (FPCs)

The functional principal component scores returned by the FPCA could be used to compare the behavior of each kinematic or acoustic variable between the two postural conditions. We carried out these comparisons by means of two-tailed paired Student’s t-tests on the eight normal (N) and constrained (SCH) score samples of each variable. The effects were considered significant for p-values equal to or less than .05, and the proportion of significance was indicated by a number of stars related to p-values: $$p<0.05^{*}, \;p<0.01^{**}, \;p<0.001^{***}$$. For the first functional behavior (referred to as *major mode*), we retained the FPC scores that significantly and directly separated the postural conditions. For the secondary functional behavior (referred to as *minor mode*), we performed a Varimax rotation of the PCA structure for prior insignificant score discrimination and retained the rotated scores if their t-test comparisons highlighted significant postural differences. Varimax rotation is a procedure of variance distribution and represents a convenient way to focus on the structure of the second variation mode to facilitate interpretation. As a consequence of this process, the *eigenfunctions* capturing the first and second behavioral differences may not be perfectly orthogonal. In practice, however, the functional units related to each of the two motor behaviors enabled clearly distinct interpretations.

#### Functional principal component regressions (FPCR)

When the FPC scores of an analysis variable could be significantly discriminated between the two postural conditions, we needed to conduct further analyses to compute the functional principal components corresponding to each intergroup variation (i.e., normal and constrained). Actually, the *eigenfunctions* returned by FPCA did not integrate the criteria for separating the postural conditions. Such a problem could be resolved by applying an inverse methodology of functional principal component regression (FPCR). This technique also allowed us to rebuild the original set of curves from the scores computed by FPCA and finally assess the fitting accuracy of our model^68,74^. Starting from a design matrix *Z* of the significant PC scores, FPCR determines *K* regression functions $\beta _{k}$ to fit at best the shape of the time series $f_i(t),i \in [1,16]$:

4 $$\begin{aligned} f_i(t)&= Z \beta (t) + \epsilon _i = \beta _0(t) + \sum _{k=1}^{K} z_{ik} \beta _{k}(t) + \epsilon _i \nonumber \\&= \beta _0(t) + \sum _{k=1}^{K} z_{ik}^{N} \beta _{k}^{N}(t) + \sum _{k=1}^{K} z_{ik}^{SCH} \beta _{k}^{SCH}(t) + \epsilon _i \nonumber \\&= \overline{f_i(t)} + \sum _{k=1}^{K} c_{ik}^{N} \xi _{k}^{N}(t) + \sum _{k=1}^{K} c_{ik}^{SCH} \xi _{k}^{SCH}(t) + \epsilon _i \end{aligned}$$

where the function $\beta _0$ corresponds to the mean curve of the time series, and $\beta _{k},k \in [1,K]$ stands for unbundled *eigenfunctions* ($\xi _{k}^{N}$ and $\xi _{k}^{SCH}$), which could not be differentiated in the FPCA context (cf Eq. 3). The score matrix *Z* enabled such separation between the two postural conditions [N/SCH] by dividing each group of eight $z_{ik}$ scores into $K=2$ columns.

#### Multiple regressions and correlations of FPC scores

Standard statistical techniques were used to highlight the main functional units shared by the cellists between the normal and constrained conditions. We determined how their motor coordination influenced the variations in bow velocity by carrying out two multivariate linear regressions, one for each functional principal component. In this design, the significant FPC scores of anatomic angles were considered predictors of the bow velocity FPC scores. This approach resulted in two models of functional motor units, which are presented in the bottom right part of Figs. 3e and 4e. Each model is also characterized by a linear relationship (cf Eqs. 1 and 2) between the most significant anatomic variables involved in the coordination chain.

Two kinds of correlation analyses were finally performed in both motion and acoustic domains. First, we extracted the important joint coupling chains of motor coordination by means of crossed correlations between the significant anatomic FPC scores. Second, we assessed functional sound-gesture interactions by computing standard Pearson correlations between the FPC scores of bow velocity and those of each acoustic descriptor. The most relevant correlations of these analyses provided a better understanding of how cellists’ motor programs influence their functional sound features in subtle ways.

## Supplementary information

Supplementary Information.

## Data Availability

The datasets generated and analyzed during the current study are available from the corresponding author on reasonable request.
