# Supplementary material for: Cellists’ sound quality is shaped by their primary postural behavior
Source: Sci Rep. 2020 Aug 17;10:13882. doi: 10.1038/s41598-020-70705-8 (PMC7431865; doi:10.1038/s41598-020-70705-8)
Supplement: Supplementary file 1 — Supplementary Information. [file 41598_2020_70705_MOESM1_ESM.pdf]

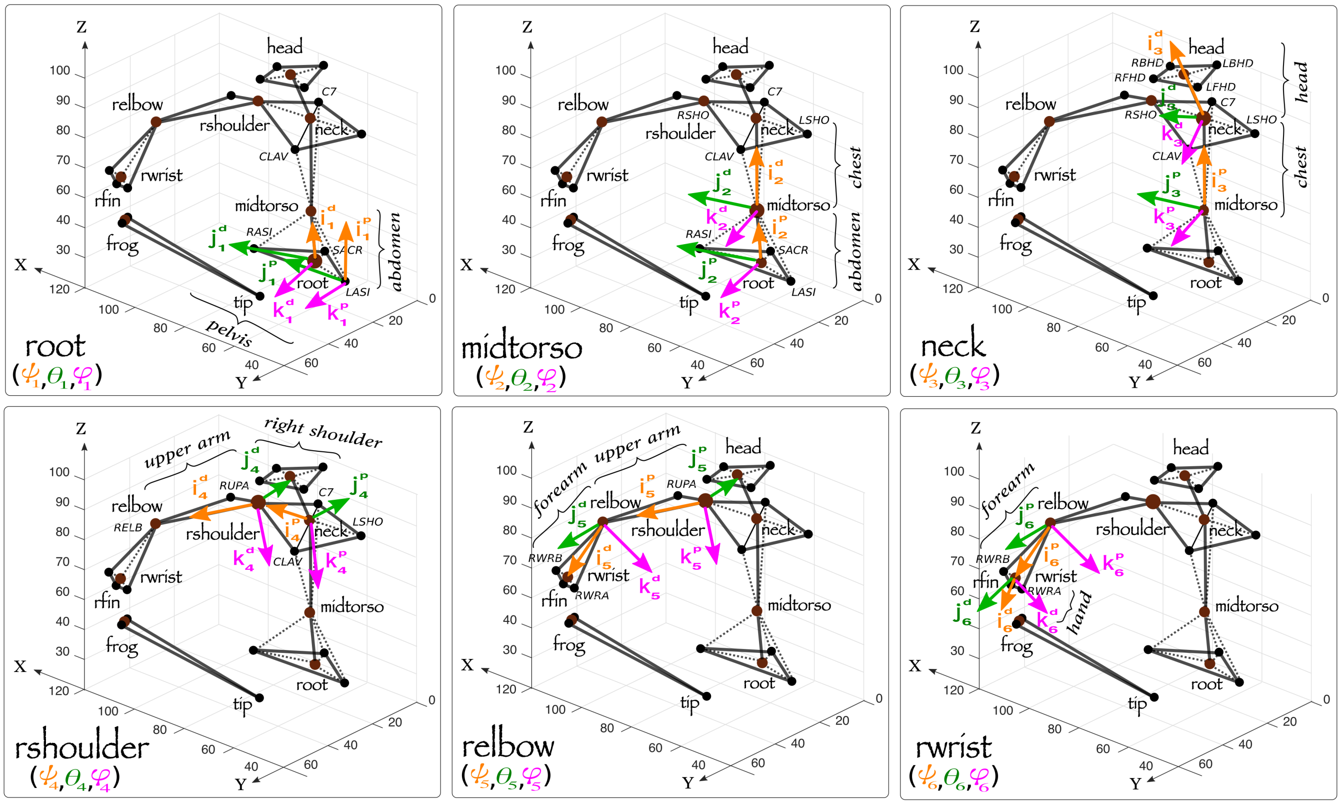

Supplementary Figure 1 | The six computational steps of cellists' joint kinematic analyses. Each step models a key rotary joint (*root, midtorso, neck, rshoulder, relbow, rwrist*) as a tridimensional rotation in the Cardan/Euler angle representation {roll (ψ_n), pitch (θ_n), yaw (ϕ_n)} where *n* is the key joint number (1…6). The proximal and distal basis of a key rotary joint are defined as triplets of three unitary vectors $\{\vec{i},\vec{j},\vec{k}\}$ representing segment-local coordinate systems (LCS) related to the joint. Joint-related euler angles are computed by three successive rotations transforming the vectors of the joint proximal basis into those of the joint distal basis (cf Supplementary Table 2): [$\psi_n : i_n^p \rightarrow i_n^d$] ; [$\theta_n : j_n^p \rightarrow j_n^d$] ; [$\phi_n : k_n^p \rightarrow k_n^d$] with *p/d* denoting the proximal and distal basis respectively.

Supplementary Table 1 | Set of markers used in the study. The key markers composing the model were obtained from one or several markers of the Plug-in-Gait standard. In this latter case, the key marker was virtual and computed as a barycenter of the related Plug-in-Gait markers.

Plug-in-gait markers	
Name	Description
LASI	Left Anterior Superior Iliac Spine
RASI	Right Anterior Superior Iliac Spine
SACR	Sacrum
CLAV	Clavicle
C7	7th Cervical vertebrae
LSHO	Left Shoulder
RSHO	Right Shoulder
LFHD	Left Front Head
RFHD	Right Front Head
LBHD	Left Back Head
RBHD	Right Back Head
RUPA	Right Upper Arm
RELB	Right Elbow
RWRA	Right Wrist A (inner side)
RWRB	Right Wrist B (outer side)
RFIN	Right Finger
FROGA	Bow Frog A (inner side)
FROGB	Bow Frog B (outer side)
TIP	Bow Tip
Key markers of the kinematical model	
Name	Related plug-in-gait markers
**root (1)**	LASI-RASI-SACR
**midtorso (2)**	CLAV-C7-LASI-RASI-SACR
**neck (3)**	LSHO-RSHO-C7
**head**	LFHD-RFHD-RBHD-LBHD
**rshoulder (4)**	RSHO
**relbow (5)**	RELB
**rwrist (6)**	RWRA-RWRB
**rfin**	RFIN
**frog (7)**	FROGA-FROGB
**tip**	TIP

Supplementary Table 2 | Proximal and distal basis used for the computation of each joint-related Euler angle. Each basis is defined as a triplet of unit vectors $\{\mathbf{i}_n^{p/d}, \mathbf{j}_n^{p/d}, \mathbf{k}_n^{p/d}\}$ where n is the key joint number $(1\ldots6)$ and *p/d* is the basis type (proximal/distal) of the joint. Please refer to Supplementary Figure 1 for the mapping between these basis vectors and the joint-related Euler angles.

Joint basis			Description of basis axes	Joint basis			Description of basis axes
**root (1)**	*Proximal*	***pelvis***	$\vec{i}_1^p = \overrightarrow{\text{LASI\_SACR}} \wedge \vec{j}_1^p$<br>$\vec{j}_1^p = \overrightarrow{\text{LASI\_RASI}}$<br>$\vec{k}_1^p = \vec{i}_1^p \wedge \vec{j}_1^p$	**rshoulder (4)**	*Proximal*	***right shoulder***	$\vec{i}_4^p = \overrightarrow{\text{neck\_rshoulder}}$<br>$\vec{j}_4^p = \vec{k}_4^p \wedge \vec{i}_4^p$<br>$\vec{k}_4^p = \vec{i}_4^p \wedge \overrightarrow{\text{neck\_C7}}$
	Distal	***abdomen***	$\vec{i}_1^d = \overrightarrow{\text{root\_midtorso}}$<br>$\vec{j}_1^d = \vec{i}_1^d \wedge \overrightarrow{\text{root\_SACR}}$<br>$\vec{k}_1^d = \vec{i}_1^d \wedge \vec{j}_1^d$		*Distal*	***upper arm***	$\vec{i}_4^d = \overrightarrow{\text{rshoulder\_relbow}}$<br>$\vec{j}_4^d = \vec{k}_4^d \wedge \vec{i}_4^d$<br>$\vec{k}_4^d = \vec{i}_4^p \wedge \overrightarrow{\text{rshoulder\_RUPA}}$
**midtorso (2)**	*Proximal*	***abdomen***	$\vec{i}_2^p = \overrightarrow{\text{root\_midtorso}}$<br>$\vec{j}_2^p = \vec{i}_2^p \wedge \overrightarrow{\text{root\_SACR}}$<br>$\vec{k}_2^p = \vec{i}_2^p \wedge \vec{j}_2^p$	**relbow (5)**	*Proximal*	***upper arm***	$\vec{i}_5^p = \overrightarrow{\text{rshoulder\_relbow}}$<br>$\vec{j}_5^p = \vec{k}_5^p \wedge \vec{i}_5^p$<br>$\vec{k}_5^p = \vec{i}_5^p \wedge \overrightarrow{\text{rshoulder\_RUPA}}$
	Distal	***chest***	$\vec{i}_2^d = \overrightarrow{\text{midtorso\_neck}}$<br>$\vec{j}_2^d = \vec{i}_2^d \wedge \overrightarrow{\text{midtorso\_C7}}$<br>$\vec{k}_2^d = \vec{i}_2^d \wedge \vec{j}_2^d$		*Distal*	***forearm***	$\vec{i}_5^d = \overrightarrow{\text{relbow\_rwrist}}$<br>$\vec{j}_5^d = \vec{i}_5^p \wedge \overrightarrow{\text{relbow\_RWRB}}$<br>$\vec{k}_5^d = \vec{i}_5^d \wedge \vec{j}_5^d$
**neck (3)**	*Proximal*	***chest***	$\vec{i}_3^p = \overrightarrow{\text{midtorso\_neck}}$<br>$\vec{j}_3^p = \vec{i}_3^p \wedge \overrightarrow{\text{midtorso\_C7}}$<br>$\vec{k}_3^p = \vec{i}_3^p \wedge \vec{j}_3^p$	**rwrist (6)**	*Proximal*	***forearm***	$\vec{i}_6^p = \overrightarrow{\text{relbow\_rwrist}}$<br>$\vec{j}_6^p = \vec{i}_6^p \wedge \overrightarrow{\text{relbow\_RWRB}}$<br>$\vec{k}_6^p = \vec{i}_6^d \wedge \vec{j}_6^d$
	Distal	***head***	$\vec{i}_3^d = \overrightarrow{\text{neck\_head}}$<br>$\vec{j}_3^d = \vec{i}_3^d \wedge \overrightarrow{\text{neck\_(LBHD+RBHD)/2}}$<br>$\vec{k}_3^d = \vec{i}_3^d \wedge \vec{j}_3^d$		*Distal*	***hand***	$\vec{i}_6^d = \overrightarrow{\text{rwrist\_RFIN}}$<br>$\vec{j}_6^p = \vec{i}_6^p \wedge \overrightarrow{\text{rwrist\_RWRB}}$<br>$\vec{k}_6^d = \vec{i}_6^d \wedge \vec{j}_6^d$
**Postural basis**				**Instrumental basis**			
